# Phenotype fingerprinting of bipolar disorder prodrome

**DOI:** 10.1186/s40345-023-00298-4

**Published:** 2023-05-18

**Authors:** Yijun Shao, Yan Cheng, Srikanth Gottipati, Qing Zeng-Treitler

**Affiliations:** 1 Washington DC VA Medical Center, Washington, DC USA; 2 Biomedical Informatics Center, George Washington University, 2600 Virginia Ave NW, Suite 300, Washington, DC 20037 USA

**Keywords:** Bipolar disorder, Phenotype, Unsupervised machine learning

## Abstract

**Background:**

Detecting prodromal symptoms of bipolar disorder (BD) has garnered significant attention in recent research, as early intervention could potentially improve therapeutic efficacy and improve patient outcomes. The heterogeneous nature of the prodromal phase in BD, however, poses considerable challenges for investigators. Our study aimed to identify distinct prodromal phenotypes or "fingerprints" in patients diagnosed with BD and subsequently examine correlations between these fingerprints and relevant clinical outcomes.

**Methods:**

20,000 veterans diagnosed with BD were randomly selected for this study. K-means clustering analysis was performed on temporal graphs of the clinical features of each patient. We applied what we call “temporal blurring” to each patient image in order to allow clustering to focus on the clinical features, and not cluster patients based upon their varying temporal patterns in diagnosis, which lead to the desired types of clusters. We evaluated several outcomes including mortality rate, hospitalization rate, mean number of hospitalizations, mean length of stay, and the occurrence of a psychosis diagnosis within one year following the initial BD diagnosis. To determine the statistical significance of the observed differences for each outcome, we conducted appropriate tests, such as ANOVA or Chi-square.

**Results:**

Our analysis yielded 8 clusters which appear to represent distinct phenotypes with differing clinical attributes. Each of these clusters also has statistically significant differences across all outcomes (p < 0.0001). The clinical features in many of the clusters were consistent with findings in the literature concerning prodromal symptoms in patients with BD. One cluster, notably characterized by patients lacking discernible prodromal symptoms, exhibited the most favorable results across all measured outcomes.

**Conclusion:**

Our study successfully identified distinct prodromal phenotypes in patients diagnosed with BD. We also found that these distinct prodromal phenotypes are associated with different clinical outcomes.

**Supplementary Information:**

The online version contains supplementary material available at 10.1186/s40345-023-00298-4.

## Introduction

Bipolar disorder (BD) is a serious mental illness, which can have significant socioeconomic and personal impacts. Mortality and suicide rate are increased in BD patients (Ketter 2010) with an estimated 30-fold increase suicide risk and a life expectancy of 8.5–9 years shorter when compared to the general population (Suicides 2017; Crump et al. 2013). Furthermore, the hospitalization rate among BD patients is much higher compared to all other patients with behavioral health diagnoses (Burden of Mental Illness 2017). Accurate and timely diagnosis of BD is important for the improvement of patient outcomes, since the failure to identify cases at early stage could prompt inadequate or inappropriate treatments, further leading to adverse consequences (Scott and Leboyer 2011). The diagnosis and treatment of BD, however, can be challenging due to the dynamic, chronic, and fluctuating nature of BD (Jann 2014).

Prodromal symptoms refer to the initial signs and symptoms that might be observed or experienced prior to the first bipolar mood episode (Meter et al. 2016; Howes et al. 2011). These symptoms commonly include mood swings, mood lability, and depression. The prodromal phase varies in clinical presentation, but much work has been done in an effort to describe it. The weighted average age for initial prodromal symptoms was 18.91 years in a study that involved 8,014 participants (Alvarez-Cadenas et al. 2023). Another article reported that as many as “50–80% of adolescents experienced depressive symptoms before the onset of the disorder (Huang et al. 2017). A meta-analysis of symptom prevalence prior to initial or recurrent mood episodes identified 11 studies (n = 1078) (Meter et al. 2016). This analysis argued that “the initial prodromal period is sufficiently long and characterized by symptoms of the subsequent mood episode to make early identification and intervention programs feasible.” At the same time, other review papers pointed to the difficulty of accurate characterization of the bipolar disorder prodrome (Howes et al. 2011; Malhi et al. 2014). The papers reported that inconsistent methods were used to assess symptoms, including different measures of screening, various time periods, and muddling self described with collateral descriptions of symptoms. Additionally, some patients did not experience prodromal symptoms prior to their diagnosis (Meter et al. 2016). The results showed a better predictor to identify BD patients is to consider symptom load, where several symptoms are experienced during a time period (Howes et al. 2011). Given the limited accurate information available on prodromal symptoms, the current understanding of prodrome is not sufficient to predict the occurrence or outcome of BD.

BD researchers have advocated the use of ‘big data’ approaches, amongst other things, to analyze the vast and complex scope of phenotypes (McIntyre et al. 2014). We hypothesize that there exist different phenotypes among prodromal symptoms, and that these phenotypes are associated with different prognoses. The specific outcomes of interest are mortality, and hospitalization rates, number of mean hospitalizations, and mean length of stay. In this study, we set out to create prodromal phenotype fingerprints for a large number of BD patients (n = 20,000) and to find links between prodrome and health outcomes. To the best of our knowledge, prior research has not attempted to identify distinct prodromal phenotypes or "fingerprints" through clustering analysis.

In the creation of prodromal phenotypes, we need to consider both known BD symptoms and moderating factors such as comorbid conditions. For instance, certain BD symptoms overlap with unipolar depression, substance abuse and schizophrenia. While certain individual symptoms could be highly specific to BD, the occurrence and co-occurrence of a group of symptoms allow a better characterization of the BD prodrome. Another consideration in phenotyping is the temporal nature of BD symptoms: the onset and duration are important features in the analysis of prodrome. A third consideration of this study is that the prodrome fingerprints should be clinically meaningful in that they are associated with varying clinical outcomes.

## Methods

### Dataset

The data source is the U.S. Veterans Affairs (VA) Corporate Data Warehouse (CDW), a large database containing the EHR data of the U.S. Veterans receiving care from the Veterans Health Administration (VHA). VHA is the largest integrated health care system in the U.S., providing outpatient and inpatient facilities across the nation. VHA offers primary and specialty services such as home health, women’s health, and mental health care. The CDW is administered by VA Informatics and Computing Infrastructure (VINCI), who provides a secure environment for researchers to access Veterans health data.

We first identified BD patients with 2 or more International Classification of Diseases (ICD) codes of BD (ICD 9 codes: 296.4X, 296.5X, 296.6X, 296.7, 296.80, and 296.89; ICD 10 codes: F31.X (PsychCentral)) in the CDW database (n = 346,511). We required 2 or more BD diagnosis codes to minimize the chance of misclassification. The dataset was further restricted to patients with (1) first BD diagnosis dated between 01/01/2001 and 09/30/2015; (2) evidence of being in the VA healthcare system ≥ 12 months prior to the first BD diagnosis; and (3) evidence of being in the VA healthcare system ≥ 12 months after the first BD diagnosis. The refined dataset has 207,838 patients. From the refined dataset, we randomly selected 20,000 patients for the prodrome fingerprinting analysis. The cohort selection is shown in Additional file 1:  Appendix I.

### Features

We experimented with large number of potential features including hospitalizations, diagnoses, procedures, medications, note types, vital signs, lab results and BD symptoms. In the identification of BD symptoms, we explored both topic modeling (Shao et al. 2015) and keyword extraction. Note types were excluded as they were often correlated with diagnoses. Thus, for the phenotype fingerprinting effort, we focused on hospitalizations, diagnoses, medications, vitals, lab results and BD symptoms. We also leveraged existing terminologies and instruments to group individual data items into groups or themes. *Hospitalization*: the beginning and end of a hospitalization is defined by the admission and discharge data from the hospitalization table. *Diagnosis groups*: We used both primary and secondary diagnostic ICD 9 codes. The ICD codes were collapsed to the first level categories (e.g., cardiovascular system disorder, etc.) Given our focus on BD, the mental illness group included one additional level of details (e.g., dementias, alcohol-induced mental disorders, etc.) (Free and ICD-9-CM Medical Coding Reference, http://www.icd9data.com). *Current Procedural Terminology (CPT) group*s: We grouped the CPT codes into seven categories including anesthesia, surgery, radiology, pathology and laboratory, evaluation and management, medicine, and HCPCS level II (Intro To and Coding: Medical Billing Codig Certification 2017). *Lab results*: The top 10 most frequent labs were used as the features, which included glucose, hemoglobin, erythrocytes, creatinine, leukocytes, carbon dioxide, potassium, sodium, chloride, and urea nitrogen. For each lab, according to the value distribution among the 20,000 patients, we standardized it into a score in the range 0 to 1, with 0 meaning the population mean and higher score meaning farther away from the population mean. *Vital signs*: As for the vital signs, we focused on blood pressure, pain score, pulse rate, body temperature, and body mass index (BMI). For each vital sign, we set standardize score as 0 for the value in the normal range, 1 for the extreme value, and unified the score in the range of > 0 and < 1 for other values. *BD symptoms*: These features were obtained from unstructured text data. Because the documentation of BD symptoms prior to diagnosis are not systematic, our initial unsupervised topic modeling effort yielded only a few topics directly associated with BD. We then identified a set of instruments that measure BD symptoms and compiled a list of symptom keywords. The instruments included depression (HAM-D), mania (YMRS, HAM-D), sleep (MADRS), psychosis (PANSS), drug-induced movement disorders (SAS, BARS, and AIMS), and suicide risk (C-SSRS), etc. (POSITIVE AND NEGATIVE SYNDROME SCALE; Montgomery-Åsberg Depression Rating Scale (MADRS); Young et al. 2000; Hamilton 1960; Barnes 1989; Simpson and Angus 1970; Rush 2000; Posner et al. 2009) The keywords were manually grouped into "BD symptoms" based on how they were labeled in the instruments. The presence of a BD symptom was determined by the presence of a keyword representing the symptom in the notes.

### Temporal data representation

**Fig. 1:**
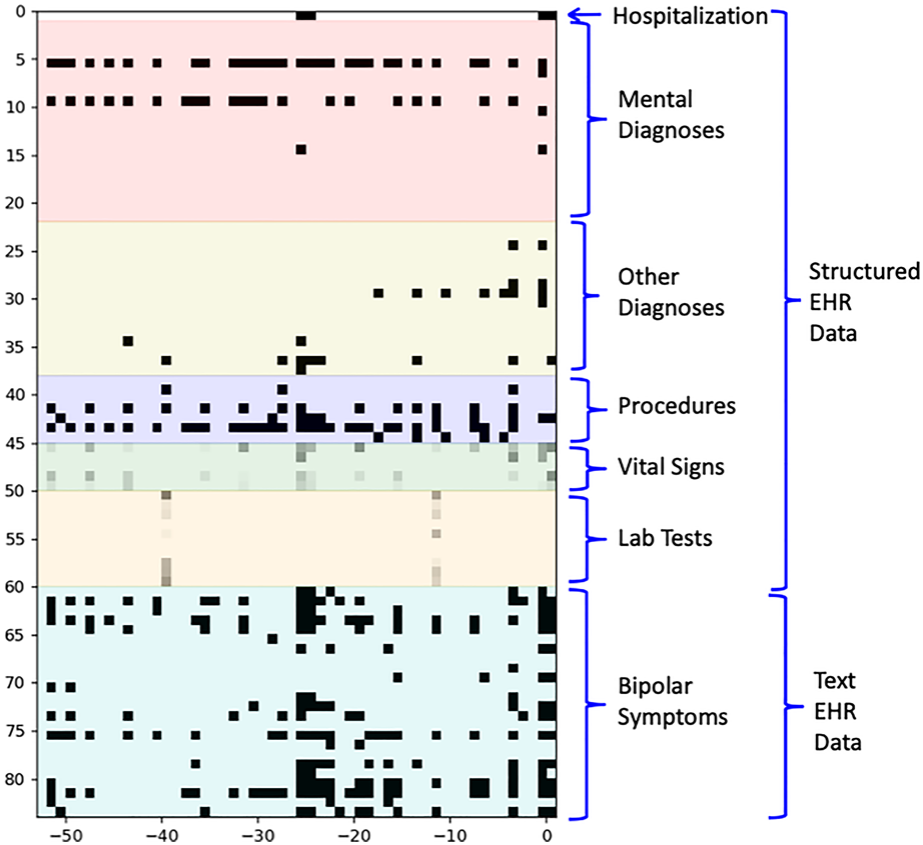
Temporal data representation from an individual patient

To represent the onset and duration of features as well as potential temporal relationships (e.g., co-occurrence), the features were represented in a temporal image. The x axis of image represents the time of the feature being reported, measured in weeks. In this study, we included 1-year data prior to the first diagnosis, so there are 52 weeks prior to the diagnosis. We also included the data during the week of the first diagnosis. In most cases, the Y axis represents the presence or absence of the feature (e.g. diagnosis). Vitals and lab results are represented using grey scale based on the deviation from the normal range (the darker the more extreme abnormality). Figure 1 is an example of temporal data representation from an individual patient. On the x-axis, 0 is the index week of BD diagnosis. Scale with minus sign means the time before the index week. For example, “-50” indicates the week of 50 before the index. On the y-axis, the numbers are the identifier of features with the order as shown in Additional file 1: Appendix II. For example, 1 is the identifier for hospitalization, 30 is ICD diagnosis of Disease of Digestive System (520–579).

### Clustering analysis

**Fig. 2:**
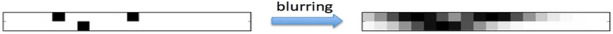
The temporal blurring operation

We performed K-means clustering analyses of the temporal images. K-means is a simple and fast unsupervised machine learning algorithm that groups a dataset into a pre-defined number (k) of clusters. It requires choosing a distance metric on the dataset: more similar pairs should have smaller distances. We chose Euclidean distance, but it was not applied directly on the temporal images: an operation which we call “temporal blurring” was first applied to the images, and then the Euclidean distances on the “blurred” images were calculated. The temporal blurring was mathematically defined as follows: let $A$ denote the matrix of the temporal image, then the blurring produces a matrix $B$ of the same size with

$$B(i,j)={max}_{j-10\le k\le j+10} A(i,k){e}^{-\frac{{(k-j)}^{2}}{10}}$$

where $A(i,k)$ is the element of $A$ in $i$th row and $k$th column, and similarly $B(i,j)$ is the element of $B$ in $i$th row and $j$th column. The effect of this operation was blurring in the time direction (Fig. 2).

The reason for the temporal blurring was from clinical consideration. With Euclidean distance, two patients who have the same diagnosis at two different times have the same distance as two patients with different diagnoses (whether at the same or different times). However, the former two patients should be more similar clinically than the latter two patients, and the similarity of the former two patients should be higher if the two times are closer. When Euclidean distance was applied to the blurred image instead of the original, the desired similarity can be achieved.

**Fig. 3:**
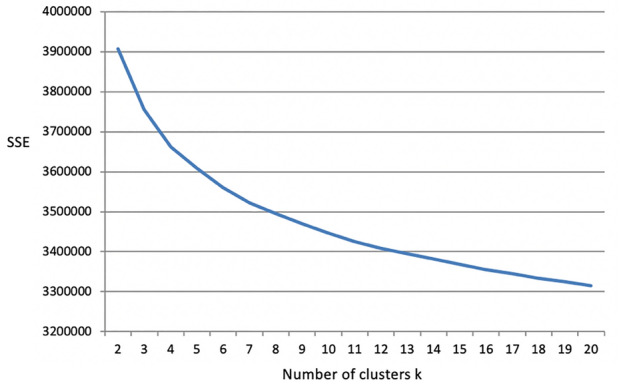
K-Means analysis to determine appropriate number of clusters

One challenge in K-means analysis is the determination of the appropriate number of clusters (i.e., k). Although there exist many mathematical tools for this problem (Everitt et al. 2011), different tools often produce inconsistent results, and none of them can work universally (Ünlü and Xanthopoulos 2019; Akhanli and Hennig 2020). Therefore, we treated the determination of k more as a clinical problem rather than a pure mathematical problem. As a clinical problem, the clusters need to be clinically reasonable, which can be validated through analysis of the clustering results. However, we still needed some initial guidance from a mathematical tool, for which we chose the elbow method, as it is simple but also widely used. We gradually increased the number of k from 2 to 20 and calculated the sum of squared errors (SSE) for the corresponding clustering result (Fig. 3). The SSE curve did not show a clear "elbow point", but we observed that SSE decreased fast initially (k = 2 to 6), then slower (k = 6 to 10), and eventually steadily in a linear decreasing manner (k = 10 to 20). Therefore, rather than looking for a single "elbow point", we considered the "elbow area", which appeared to be between 6 and 10. Finally, we chose k = 8, but note that this choice was not considered as the only "correct" answer, as the nearby k values may also be good choices. For each cluster, the mean temporal image, whose pixel values were the mean of the corresponding pixels in the original temporal images within the cluster, was used as the *phenotype fingerprint* of the cluster. The clinical reasonableness of this clustering result was validated in the next step as described below.

### Associated clinical outcomes

We calculated the mortality rate, hospitalization rate, mean number of hospitalizations, mean length of stay and psychosis diagnosis with 1 year after the diagnosis. The mean age and gender distribution of each cluster was calculated. We also collected data on the type of medications received by each cluster. On each of outcomes, statistical testing (ANOVA or Chi-square test) was done for the significance of their differences. Considering the large sample size would cause significant p-value even for tiny differences between groups, we also calculated the absolute standardized difference (ASD) for each of the other clusters compared to Cluster 1 (reference group), for which a value of 10% or above indicated the difference between the two groups (Austin 2009).

## Results

### Dataset

Among 20,000 randomly selected BD patients, 84.2% were male, 73.4% were White, 17.2% were Black, and 5.4% were Hispanics (Table 1). The cohort were relatively young with the mean age of 49 years old.

**Table 1:** Cohort demographic characteristics

Demographics	Mean/N	STD/%
Age (Years)	48.8	13.5
Male	16,842	84.2%
Female	3,158	15.8%
Race White	14,673	73.4%
Race Black	3,434	17.2%
Race Others	395	2.0%
Race Unknown	1,498	7.5%
Ethnicity Hispanics	1,072	5.4%
Ethnicity Non-Hisapnics	17,896	89.5%
Ethnicity Unknown	1,032	5.2%

STD, standard deviation

### Clustering analysis

**Fig. 4:**
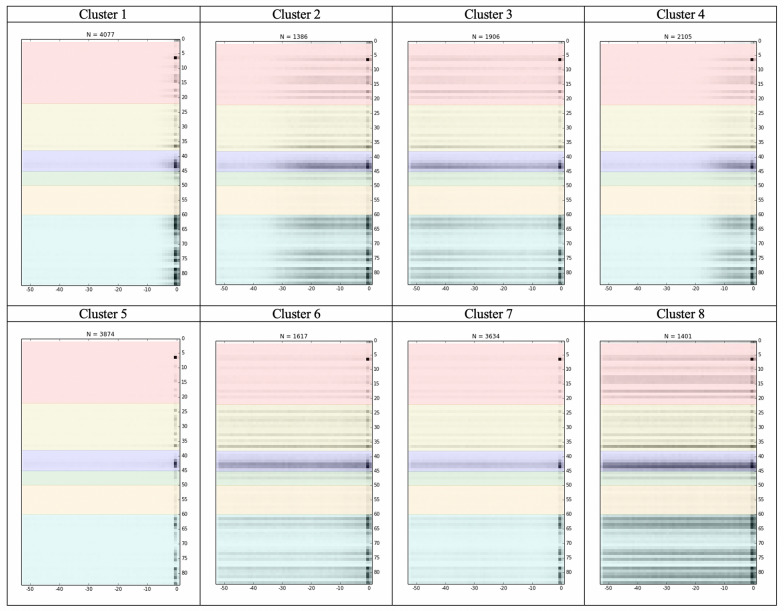
Aggregated image of temporal data from each of eight clusters

The patients were grouped into 8 clusters. We aggregated the temporal data representation of prodrome for patients in each cluster into a single image, which resulted in 8 images shown in Fig. 4. We also tried different cluster numbers varying from 2 to 10 to group patients. The aggregate images of temporal features were shown in Additional file 1: Appendix III (A1–A8).

### Patient baseline characteristics comparison across clusters

**Fig. 5:**
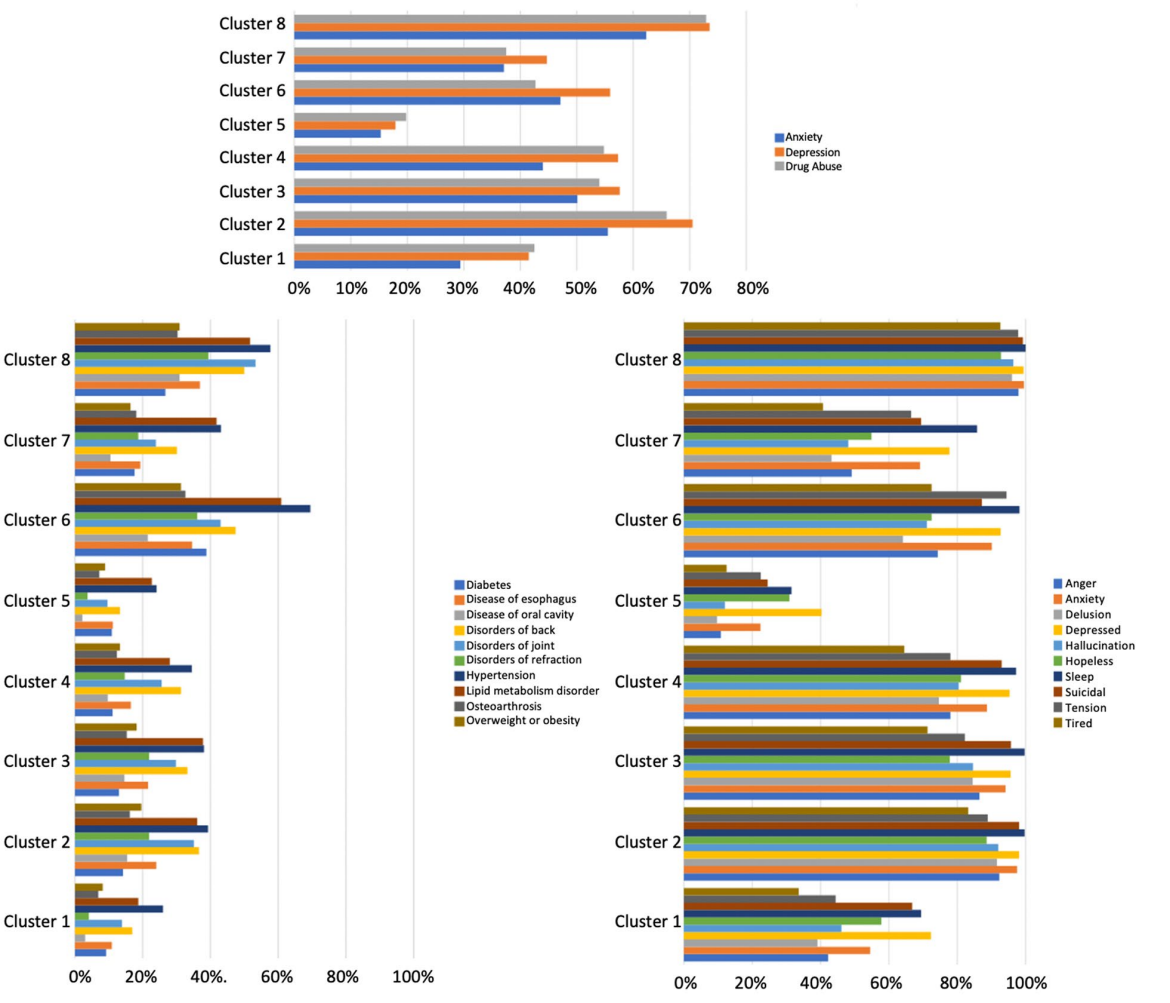
Top mental disorders, bipolar disorder symptoms, and comorbid conditions before initial bipolar disorder diagnosis

We compared patient characteristics in Table 2 and Fig. 5 (detailed information for Fig. 5 were summarized in Additional file 1: Appendix IV-VI). Patients in Clusters 1 and 5 had the shortest prodromal period than others (Fig. 4). These two clusters shared similar characteristics with the other clusters, including younger ages (45 and 49 years), higher proportions of females (16.8% and 16.7%), lower proportions of Hispanics (4.8% and 4.1%), lower prevalence rates of anxiety (29.4% and 15.3%) and all the comorbid conditions listed in Fig. 5 and Additional file 1: Appendix V. However, the main differences between patients in Clusters 1 and 5 existed in higher prevalence of mental disorders and BD symptoms among patients in Cluster 1 than those in Cluster 5, such as drug abuse (42.5% vs 19.8%), depression (41.5% vs 17.9%), and anxiety (29.4% vs 15.3%) (Fig. 5 and Additional file 1: Appendix IV and VI).

**Table 2:** Demographic characteristics across clusters

	Cluster 1 N = 4077	Cluster 2 N = 1386	Cluster 3 N = 1906	Cluster 4 N = 2105	Cluster 5 N = 3874	Cluster 6 N = 1617	Cluster 7 N = 3634	Cluster 8 N = 1401
Age, Mean (STD)	45.1 (13.5)	46.2 (12.6)	48.4 (12.6)	46.5 (13.1)	49.1 (14.4)	55.6 (12.1)	51.5 (13.1)	50.8 (12.0)
Female%	16.8%	16.0%	16.0%	14.6%	16.7%	15.0%	13.9%	16.9%
Race% White	72.3%	71.6%	75.3%	73.2%	74.6%	75.1%	74.3%	68.3%
Black	18.4%	21.1%	17.6%	19.1%	12.4%	17.5%	15.2%	24.3%
Others	2.2%	1.7%	1.7%	1.2%	2.3%	1.5%	2.2%	2.5%
Unknown	7.2%	5.7%	5.5%	6.5%	10.8%	5.9%	8.3%	4.9%
Hispanics%	4.8%	6.6%	6.1%	6.0%	4.1%	5.6%	5.6%	6.4%

STD, standard deviation

Patients in Clusters 2 and 4 had a gradually intensive prodrome (Fig. 4). Compared to the other clusters, these two clusters had younger ages (46 years for both), high proportions of Blacks (21.1% and 19.1%) and Hispanics (6.6% and 6.0%). Their prevalence rates of mental disorders, BD symptoms, and comorbid conditions were higher than patients in Cluster 1 and 5 (Fig. 5 and Additional file 1: Appendix IV-VI). Besides, patients in Cluster 2 had even consistently higher rates than those in Cluster 4, such as drug abuse (65.9% vs 54.8%), depression (70.5% vs 57.3%), and anxiety (55.5% vs 44.0%).

The prodrome of patients in Clusters 3 and 6–8 started as early as at least one year before the BD diagnosis (Fig. 4). In comparison, patients in Cluster 3 had slightly decreased intensive prodrome later in the prodromal period; patients in Cluster 8 had the most intensive prodrome, followed by patients in Clusters 6 and 7. In the patients of these four clusters, patients in Cluster 3 were younger (48 years), had higher proportion of female and Hispanics (6.1%), and were healthier (lower rate of comorbid conditions, Fig. 5 and Additional file 1: Appendix V) than the other three clusters. But patients in Cluster 3 had higher rate of mental disorders (drug abuse for 54.0%, depression for 57.6%, and anxiety for 50.1%) and BD symptoms than patients in Clusters 6 and 7, but lower rate than patients in Cluster 8 (Fig. 5 and Additional file 1: Appendix IV and VI). Patients in Clusters 6 and 7 were older (56 and 52 years), with lower proportions of females (15.0% and 13.0%), Blacks (17.5% and 15.2%), and Hispanics (4.1% and 5.6%), and with the medium prevalence of mental disorders (drug abuse for 42.7% and 37.5%, depression for 55.9% and 44.7%, and anxiety 47.1% and 37.1%) than Clusters 3 and 8. However, patients in Cluster 7 were healthier than patients in Cluster 6 in terms of lower prevalence of comorbid conditions and BD symptoms (Fig. 5 and Additional file 1: Appendix V and VI). Patients in Cluster 6 had the poorest health since they had the highest prevalence of comorbid conditions among all patients (Fig. 5 and Additional file 1: Appendix V). Characteristics of patients in Cluster 8 included older age (51 years), highest proportion of female (16.9%) and Blacks (24.3%), highest prevalence of mental disorders (drug abuse 72.9%, depression 73.5%, and anxiety 62.3%) and BD symptoms, and second highest prevalence of comorbid conditions (Fig. 4 and Additional file 1: Appendix IV-VI).

### Clinical outcomes

As shown in Table 3 and Additional file 1: Appendix VII, the clusters were statistically significantly different on all outcomes (all p-values < 0.0001). This suggests that they were distinct sub patient populations. Since these clusters were created using phenotypical features, the results also suggest that these populations had different phenotypes.

**Table 3:** One-year outcomes of eight clusters

	Cluster 1 N = 4077	Cluster 2 N = 1386	Cluster 3 N = 1906	Cluster 4 N = 2105	Cluster 5 N = 3874	Cluster 6 N = 1617	Cluster 7 N = 3634	Cluster 8 N = 1401	p-value
Death%	1.3%	2.1%	0.8%	1.4%	1.0%	4.6%	1.4%	2.6%	< 0.0001
ASD (%) for death	Reference	6	5	1	3	**20**	1	9	-
Hospitalization%	27.9%	36.1%	27.3%	27.6%	13.5%	35.1%	20.0%	51.1%	< 0.0001
ASD (%) for hospitalization%	Reference	**18**	1	1	**36**	**16**	**19**	**49**	-
#Hospitalizations, Mean (SD)	0.5 (1.1)	0.8 (1.4)	0.5 (1.1)	0.6 (1.3)	0.2 (0.7)	0.7 (1.4)	0.3 (0.9)	1.4 (2.2)	< 0.0001
ASD (%) for #hospitalizations	Reference	**24**	0	8	**33**	**16**	**20**	**52**	-
Length of Stay, Mean (SD)	11.1 (35.1)	14.5 (39.3)	9.3 (30.4)	10.2 (34.0)	4.0 (21.3)	9.4 (32.2)	5.9 (26.0)	22.8 (52.9)	< 0.0001
ASD(%) for length of stay	Reference	9	5	3	**24**	5	**17**	**26**	-

ASD, absolute standardized difference; ASD ,≥ 10% indicates difference between groups; The values of ASD ≥10% are the bold.

Cluster 1 had the largest size, accounting for about 25% of the patients. This cluster was youngest and did not have significant prodromal symptoms, so we used it as the reference group. Compared to the other clusters, Cluster 1 had BD symptoms at the onset appearing more intense (Fig. 4).

The presence, length, and intensity of prodromal symptoms in each phenotype appear to be generally associated with patient outcomes. Cluster 8’s fingerprint showed the long lasting and intense prodromal symptoms (Fig. 4), highest hospitalization rate (51.1%), greated number of hospitalizations (1.4 ± 2.2), and longest hospital length of stay (22.8 ± 52.9 days) in the first year after first BD diagnosis. Cluster 5’s fingerprint showed no prodromal symptoms (Fig. 4) and had the best outcomes in the lowest hospitalization rate (13.5%), fewest number of hospitalization (0.2 ± 0.7), and shortest length of stay (4.0 ± 21.3 days). Cluster 2 had a longer prodromal period than Cluster 4 and worse outcomes (Fig. 4 and Table 3).

Comorbid conditions are an important factor. Cluster 6 did not have particularly intense prodromal symptoms but had the highest death rate (4.6%). Its fingerprint showed more diagnoses in the year before BD. The hospitalization rate, number of hospitalizations, and length of stay were also higher than some of the other clusters but are not the highest (Table 3).

As shown in Table 4 and Additional file 1: Appendix VII, the majority of hospitalizations were caused by mental illness. However, patients in Cluster 6 hospitalized mainly caused by other reasons. In comparison, patients in Cluster 7 also had lower proportion of mental illness caused hospitalization than those in other clusters. Patients in Cluster 1–3 had the highest rate of mental-illness-caused hospitalization among all patients.

**Table 4:** Outcome of hospitalizations due to mental illness

	Cluster 1	Cluster 2	Cluster 3	Cluster 4	Cluster 5	Cluster 6	Cluster 7	Cluster 8
Proportion of Hospitalizations due to Mental Illness	73.0%	71.8%	77.2%	66.2%	63.9%	35.3%	57.8%	64.1%
ASD (%) for Proportion of Hospitalizations due to Mental Illness	Reference	3	**10**	**15**	**20**	**82**	**32**	**19**
Proportion of Hospitalized Patients Having at least one hospitalization due to Mental Illness	77.3%	77.2%	78.5%	71.0%	68.9%	44.5%	60.7%	72.1%
ASD (%) for Proportion of Hospitalized Patients Having at least one hospitalization due to Mental Illness	Reference	0	3	**15**	**19**	**71**	**36**	**12**

ASD, absolute standardized difference; ASD, ≥ 10% indicates difference between groups; The values of ASD ≥10% are the bold.

## Discussion

We identified prodromal phenotype fingerprints through clustering analysis on a large set of BD patients (n = 20,000). To the best of our knowledge, prior prodromal analyses have not been performed on such a large sample size and did not characterize the different prodrome phenotypes. A systematic review and meta-analysis study reported that the sample size from the published studies ranged from 15 to 600 patients (Meter et al. 2016; Skjelstad et al. 2010). These studies primarily relied on patients’ self or family member reported data from the questionnaires or interviews. Our study is the first study that incorporated comprehensive data sources including both structured data (hospitalization history, ICDs, CPTs, labs, and vital signs) and unstructured data (BD topics identified free-text from the electronic medical records).

In this study, we found the different phenotypes were associated with statistically significantly different outcomes. For example, patients in Cluster 6 were oldest and had most prevalent comorbid conditions; as a result, the hospitalization mainly caused by other diseases than mental illness and they also had the highest death rate. Patients in Cluster 8 had most prevalent mental illness problems and higher prevalence of other comorbid conditions during prodromal period, which resulted in the highest hospitalization rate and longest length of stay. In comparison, patients in Cluster 5, with higher proportion of females than those in other clusters, were healthiest and with least prevalence of mental disorders; therefore, they had the lowest hospitalization and death rate. When incorporating prodrome fingerprint of each cluster shown in Fig. 4, we may see patients Cluster 5 were relatively healthier than all of other clusters, since they almost had no prodromal symptoms before BD diagnosis and only had observations in one week after BD diagnosis. It is very interesting to compare Cluster 3 and 6. The patients in Cluster 3 had much better outcomes than those in Cluster 6; however, Cluster 3 appeared to have more intense prodromal symptoms especial in the top (y-axis:2–22) and bottom (y-axis:61–84) part of the fingerprint, which were about mental disorder diagnosis and BD topics. One potential explanation is that they were not the key factors causing death or hospitalization, but other comorbid conditions were, because there were more intense prodromal symptoms in the top (y-axis:23–40) part of fingerprint of Cluster 6.

The prodrome fingerprints are consistent with the prior literature’s findings of prodromal symptoms in BD patients: many, but not all BD patients have a prodrome phase (Skjelstad et al. 2010; Janardhan Reddy 2012; Faedda et al. 2015). The prodromal phase of BD refers to the “time interval between the onset of the first prodromal symptom and the onset of the characteristic signs/symptoms of BD” (Huang et al. 2017). This phase has varying length and intensity, ranging from as short as 4 months to as long as 10 years (Meter et al. 2016). Previous studies have investigated the prodromal phase and developed tools to detect it, such as the person-level risk calculator (Alvarez-Cadenas et al. 2023). Likewise, Huang et al. (2017) used Twitter to conduct their research on the prodromal phase after finding that individuals often shared their prodromal symptoms on the social media platform (Huang et al. 2017). Furthermore, in this study, we found the length and intensity of symptoms appeared to be associated with clinical outcomes, which were rarely investigated in the published studies.

The important implication of the study is that prodromal patterns are associated with BD outcomes. The BD patients with the worst outcomes had either long and/or intense prodrome symptoms, or more comorbidities. Clinicians may adjust their treatment strategies according to the patients’ prodromal fingerprints.

Worth noting is that several phenotypes do not have significant prodrome symptoms or co-morbid conditions based on the medical records. Since we selected only patients with at least 1-year pre and post BD diagnosis data, these patients are in the healthcare system. It is possible that their symptoms were overlooked or not reported. It is also possible that these sub-populations have a sudden onset or these BD cases are diagnosed promptly. In fact, this may be why patients who have no prodrome tend to have better outcomes.

Admittedly, this study has some limitations. One limitation is that we examined phenotype fingerprints within a BD cohort, without a control group. In the next steps, we would like to apply the fingerprinting methods to patients without BD (but may have other forms of mental illnesses). Those patients would serve as a control population for the BD patients. Another limitation is that our BD sample contains a greater proportion of older, male patients than the general BD population and no pediatric patients. This can affect the generalizability of out findings. It has been suggested that random permutation with class balance is a possible solution for unbiased cohort selection. In future analysis, we would like to enrich the dataset by integrating it with medical records other healthcare systems.

While there is a much larger number of patients with BD in the VA system, we sampled 20,000 for the clustering analysis. One article suggested that a sample size of 20 per subgroup is sufficient to discover a “true” cluster. When the cluster separation is large (Dalmaijer et al. 2022). In multi-dimensional space, class separation however is usually quite large when measured as the distance between centroids. Indeed, on complex data, clustering analysis can be viewed as discovery of patterns, where there are more than one correct answer rather than a single “true” classification. Another publication suggested that the sample size should be 70 times of the number of features (Dolnicar et al. 2014). In this study, we had 84 features. Considering the temporal dimension, a larger sample size is desired. We chose a sample size of 20,000, which is large but still manageable in our computational environment.

## Conclusion

Phenotype fingerprint based on the temporal prodromal symptom are associated with different outcomes in patients with BD. This knowledge can assist providers in identifying patients at higher risk of adverse outcomes.

## Supplementary Information

**Additional file 1. **Appendix I-VII

## Data Availability

Not applicable.
