# Supplementary material for: Phenotype fingerprinting of bipolar disorder prodrome
Source: Int J Bipolar Disord. 2023 May 18;11:19. doi: 10.1186/s40345-023-00298-4 (PMC10195932; doi:10.1186/s40345-023-00298-4)

**Appendix I Cohort Selection**

Patients with >=2 ICD codes of bipolar disorder in the VA CDW database

(N=346,511)

Patients with first bipolar diagnosis dated between 01/01/2001 and 09/30/2015 (N=258,150)

Patients being in VA system for >=12 months prior to first bipolar diagnosis (N=209,925)

Patients being in VA system for >=12 months after first bipolar diagnosis (N=207,838)

Randomly selected N=20,000 patients

**Appendix II Features for Fingerprinting of Bipolar Disorder Prodrome**

| **Feature Group** | **ID** | **Feature Name** | **Data Points** | **Definition** |
| --- | --- | --- | --- | --- |
| Hospitalization | 1 | Hospitalization | 13,161 | Defined as an inpatient visit |
| Mental Disorder Diagnoses | 2 | Alcohol-induced mental disorders (291) | 1,865 | ICD: 291.* |
|  | 3 | Drug-induced mental disorders (292) | 2,946 | ICD: 292.* |
|  | 4 | Transient mental disorder due to conditions classified elsewhere (293) | 2,848 | ICD: 293.* |
|  | 5 | Persistent mental disorder due to conditions classified elsewhere (294) | 3,293 | ICD: 294.* |
|  | 6 | Schizophrenic disorders (295) | 34,964 | ICD: 295.* |
|  | 7 | Episodic mood disorders (296) | 66,781 | ICD: 296.* |
|  | 8 | Delusional disorders (297) | 667 | ICD: 297.* |
|  | 9 | Other nonorganic psychoses (298) | 5,891 | ICD: 298.* |
|  | 10 | Anxiety dissociative and somatoform disorders (300) | 31,002 | ICD: 300.* |
|  | 11 | Personality disorders (301) | 8,379 | ICD: 301.* |
|  | 12 | Sexual and gender identity disorders (302) | 1,434 | ICD: 302.* |
|  | 13 | Alcohol dependence syndrome (303) | 46,479 | ICD: 303.* |
|  | 14 | Drug dependence (304) | 47,788 | ICD: 304.* |
|  | 15 | Nondependent abuse of drug (305) | 41,774 | ICD: 305.* |
|  | 16 | Special symptoms or syndromes not elsewhere classified (307) | 1,746 | ICD: 307.* |
|  | 17 | Acute reaction to stress (308) | 345 | ICD: 308.* |
|  | 18 | Adjustment reaction (309) | 65,297 | ICD: 309.* |
|  | 19 | Specific nonpsychotic mental disorders due to brain damage (310) | 671 | ICD: 310.* |
|  | 20 | Depressive disorder not elsewhere classified (311) | 43,137 | ICD: 311.* |
|  | 21 | Disturbance of conduct not elsewhere classified (312) | 1,843 | ICD: 312.* |
|  | 22 | Hyperkinetic syndrome of childhood (314) | 2,584 | ICD: 314.* |
| Other Diagnoses | 23 | Infectious and parasitic diseases (001-139) | 10,920 | ICD: 001.*-139.* |
|  | 24 | Neoplasms (140-239) | 6,217 | ICD: 140.*-239.* |
|  | 25 | Endocrine, nutritional and metabolic diseases, and immunity disorders (240-279) | 39,560 | ICD: 240.*-279.* |
|  | 26 | Diseases of the blood and blood-forming organs (280-289) | 3,612 | ICD: 280.*-289.* |
|  | 27 | Diseases of the nervous system and sense organs (320-389) | 27,742 | ICD: 320.*-389.* |
|  | 28 | Diseases of the circulatory system (390-459) | 37,147 | ICD: 390.*-459.* |
|  | 29 | Diseases of the respiratory system (460-519) | 16,307 | ICD: 460.*-519.* |
|  | 30 | Diseases of the digestive system (520-579) | 25,277 | ICD: 520.*-579.* |
|  | 31 | Diseases of the genitourinary system (580-629) | 10,907 | ICD: 580.*-629.* |
|  | 32 | Diseases of the skin and subcutaneous tissue (680-709) | 9,220 | ICD: 680.*-709.* |
|  | 33 | Diseases of the musculoskeletal system and connective tissue (710-739) | 50,696 | ICD: 710.*-739.* |
|  | 34 | Congenital anomalies (740-759) | 587 | ICD: 740.*-759.* |
|  | 35 | Symptoms, signs, and ill-defined conditions (780-799) | 38,813 | ICD: 780.*-799.* |
|  | 36 | Injury and poisoning (800-999) | 11,371 | ICD: 800.*-999.* |
|  | 37 | Supplementary classification of factors influencing health status and contact with health services (V01-V91) | 175,604 | ICD: V01.*-V91.* |
|  | 38 | Supplementary classification of external causes of injury and poisoning (E000-E999) | 3,643 | ICD: E000.*-E999.* |
| Procedures | 39 | Anesthesia (00100-01999,99100-99150) | 922 | CPT: 00100-01999, 99100-99150 |
|  | 40 | Surgery (10021-69990) | 16,209 | CPT: 10021-69990 |
|  | 41 | Radiology (70010-79999) | 29,794 | CPT: 70010-79999 |
|  | 42 | Pathology and laboratory (80047-89356) | 73,856 | CPT: 80047-89356 |
|  | 43 | Evaluation and management (99201-99499) | 222,767 | CPT: 99201-99499 |
|  | 44 | Medicine (90281-99099,99151-99199,99500-99607) | 278,074 | CPT: 90281-99099,99151-99199,99500-99607 |
|  | 45 | HCPCS level ii (A0000-V9999) | 74,748 | CPT: A0000-V9999 |
| Vital Signs | 46 | Blood pressure | 171,655 | Defined using the feature name as the vital sign name from the vital sign table |
|  | 47 | BMI | 42,869 |  |
|  | 48 | Pain | 161,357 |  |
|  | 49 | Pulse | 170,653 |  |
|  | 50 | Temperature | 152,812 |  |
| Lab Results | 51 | Carbon dioxide | 47,720 | Defined using LOINC codes whose component (or  analyte) name equal the feature name |
|  | 52 | Chloride | 48,817 |  |
|  | 53 | Creatinine | 45,839 |  |
|  | 54 | Erythrocytes | 43,304 |  |
|  | 55 | Glucose | 64,075 |  |
|  | 56 | Hemoglobin | 46,422 |  |
|  | 57 | Leukocytes | 45,881 |  |
|  | 58 | Potassium | 49,595 |  |
|  | 59 | Sodium | 49,463 |  |
|  | 60 | Urea nitrogen | 48,783 |  |
| BD Symptoms | 61 | Agitation | 109,083 | Defined using a set of instruments. The presence of each symptom on a patient is defined as the presence of a symptom keyword in the notes. |
|  | 62 | Anxiety | 301,093 |  |
|  | 63 | Appearance | 154,808 |  |
|  | 64 | Depressed | 476,033 |  |
|  | 65 | Disruptive | 283,601 |  |
|  | 66 | Elevated mood | 31,155 |  |
|  | 67 | Gastro-intestinal | 86,656 |  |
|  | 68 | General somatic | 33,725 |  |
|  | 69 | Genital | 20,619 |  |
|  | 70 | Guilt | 30,815 |  |
|  | 71 | Hypochondriasis | 5,594 |  |
|  | 72 | Increased motor activity | 38,022 |  |
|  | 73 | Insight | 111,282 |  |
|  | 74 | Irritability | 251,658 |  |
|  | 75 | Loss of weight | 32,909 |  |
|  | 76 | Questionable plans | 464,227 |  |
|  | 77 | Retardation | 21,988 |  |
|  | 78 | Sexual interest | 1,745 |  |
|  | 79 | Sleep | 406,614 |  |
|  | 80 | Somatic anxiety | 106,256 |  |
|  | 81 | Speech | 200,570 |  |
|  | 82 | Suicide | 518,415 |  |
|  | 83 | Thought disorder | 53,871 |  |
|  | 84 | Work and activities | 255,935 |  |

**Appendix III Aggregated Temporal Images from the Clustering Result with Different Number of Clusters**

A1. Aggregated Temporal Images from the Clustering Result with 2 Clusters

| Cluster 1 | Cluster 2 |
| --- | --- |
| 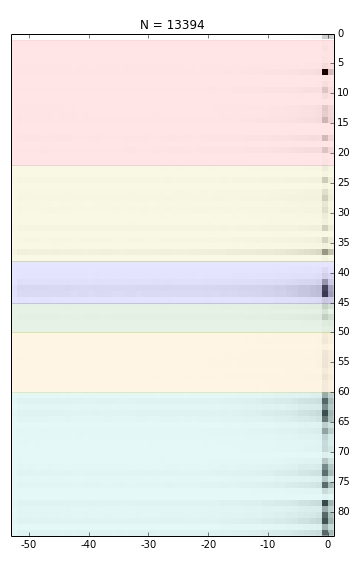 | 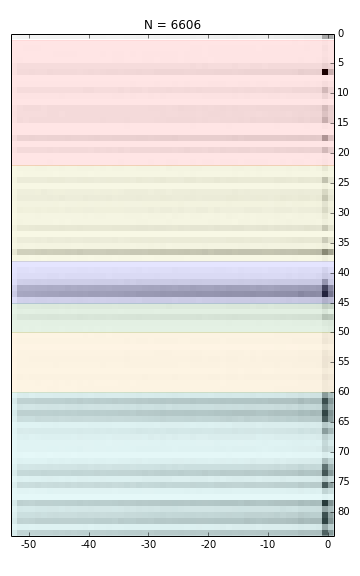 |

A2. Aggregated Temporal Images from the Clustering Result with 3 Clusters

| Cluster 1 | Cluster 2 | Cluster 3 |
| --- | --- | --- |
| 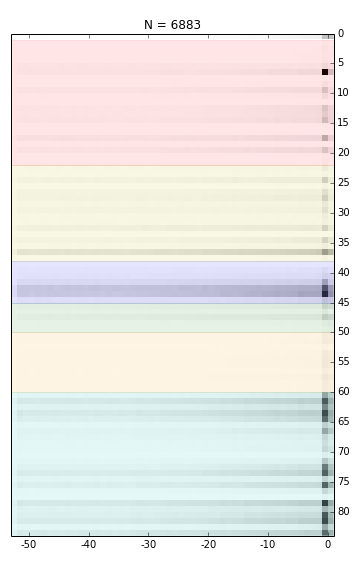 | 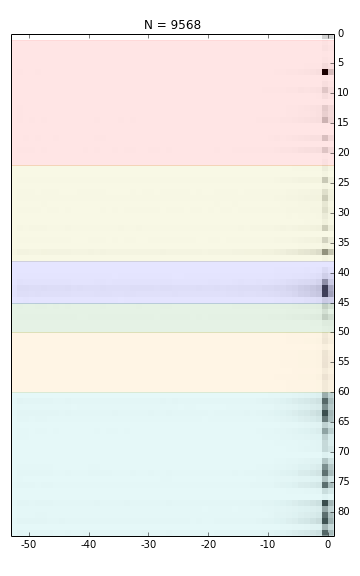 | 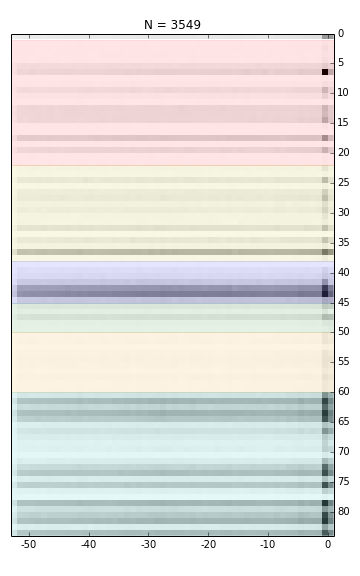 |

A3. Aggregated Temporal Images from the Clustering Result with 4 Clusters

| Cluster 1 | Cluster 2 | Cluster 3 | Cluster 4 |
| --- | --- | --- | --- |
| 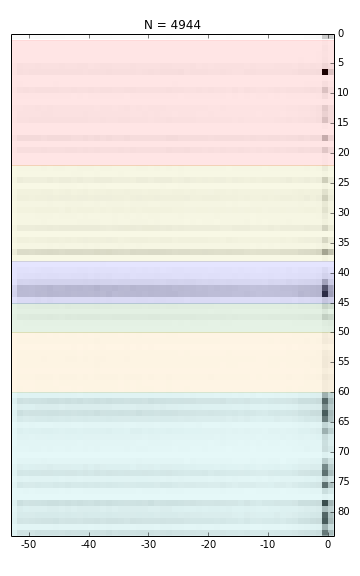 | 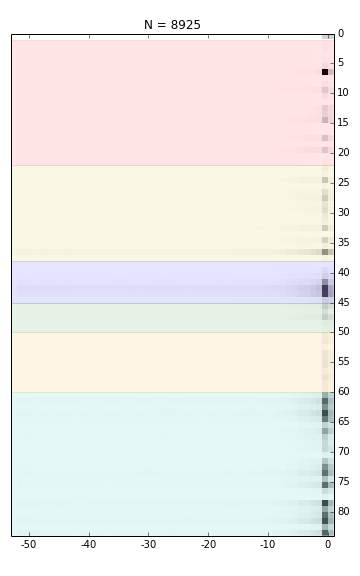 | 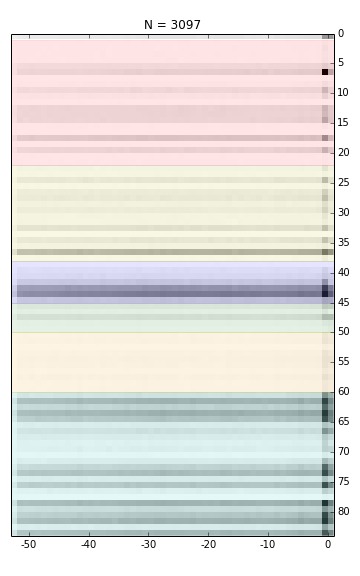 | 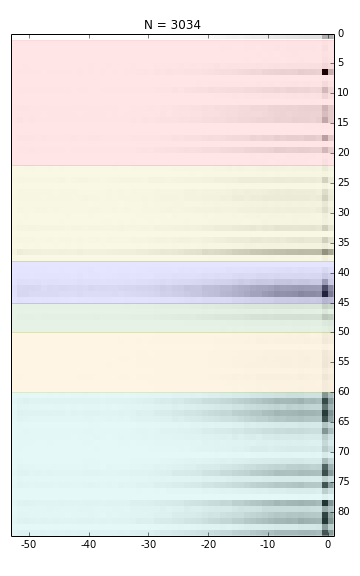 |

A4. Aggregated Temporal Images from the Clustering Result with 5 Clusters

| Cluster 1 | Cluster 2 | Cluster 3 |
| --- | --- | --- |
| 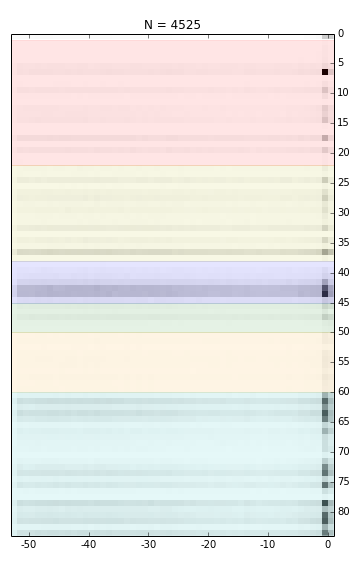 | 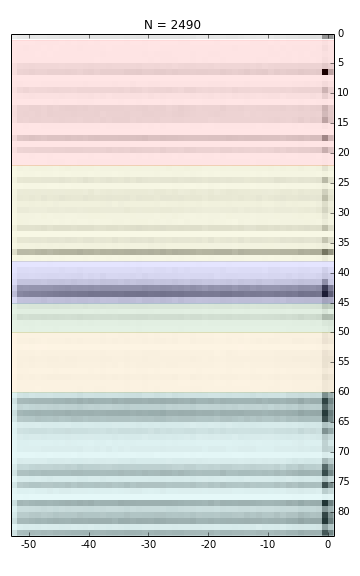 | 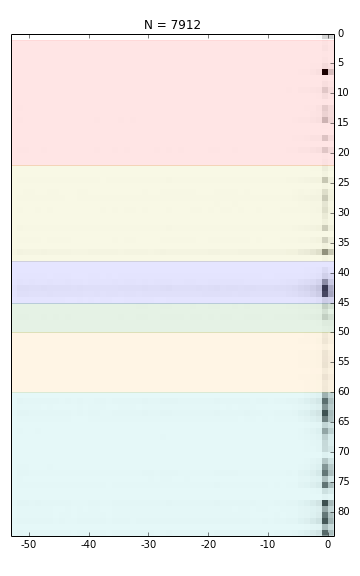 |
| Cluster 4 | Cluster 5 |  |
| 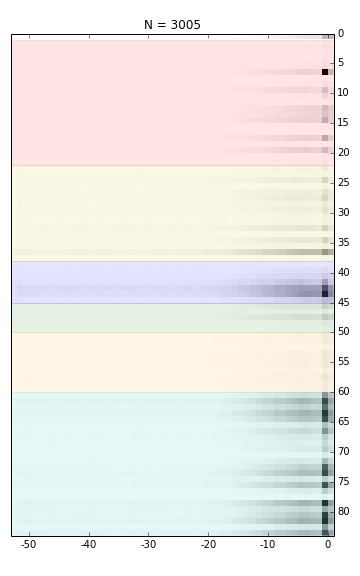 | 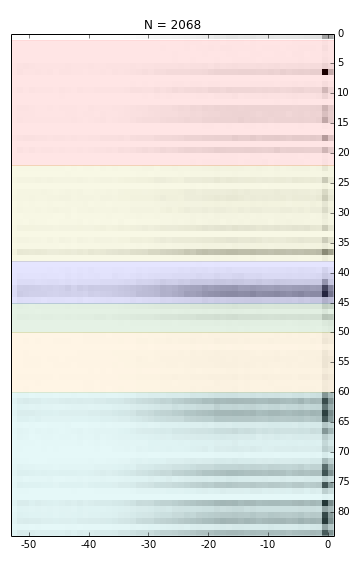 |  |

A5. Aggregated Temporal Images from the Clustering Result with 6 Clusters

| Cluster 1 | Cluster 2 | Cluster 3 |
| --- | --- | --- |
| 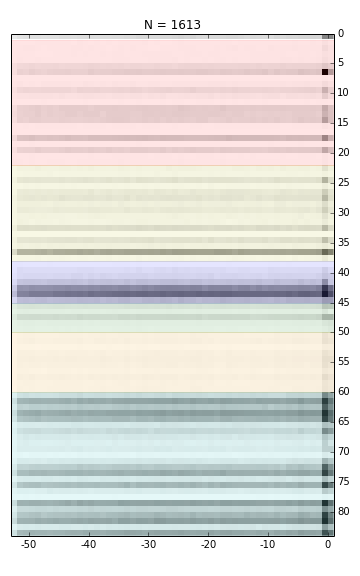 | 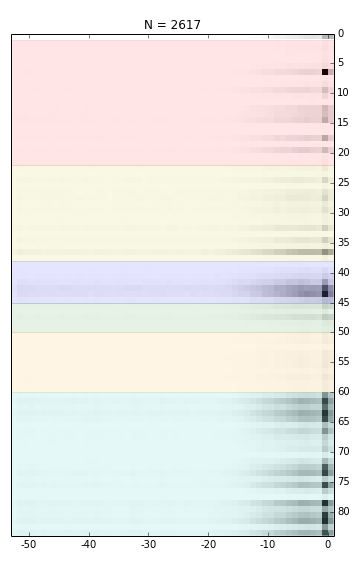 | 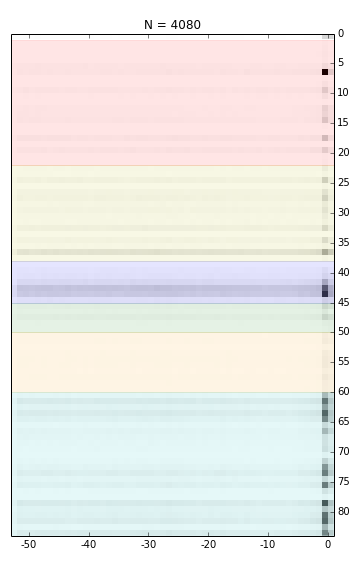 |
| Cluster 4 | Cluster 5 | Cluster 6 |
| 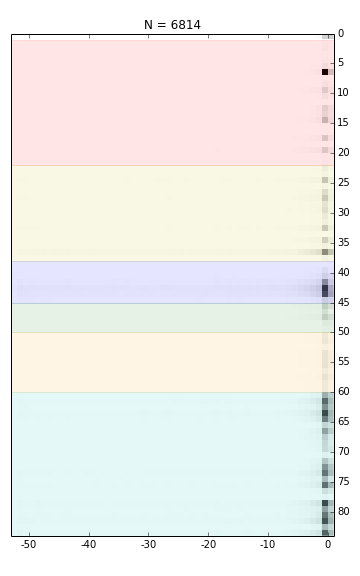 | 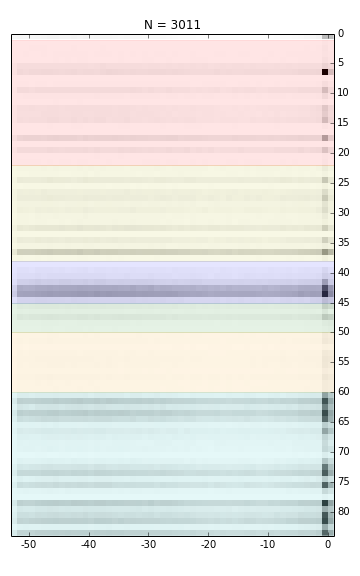 | 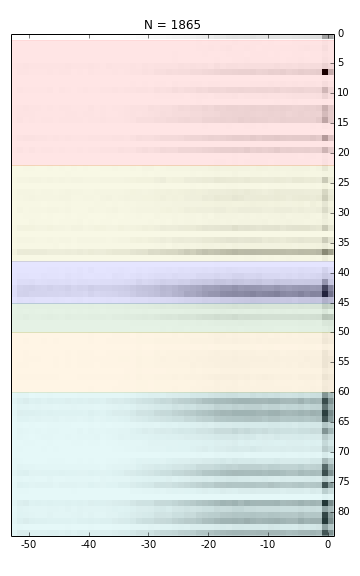 |

A6. Aggregated Temporal Images from the Clustering Result with 7 Clusters

| Cluster 1 | Cluster 2 | Cluster 3 | Cluster 4 |
| --- | --- | --- | --- |
| 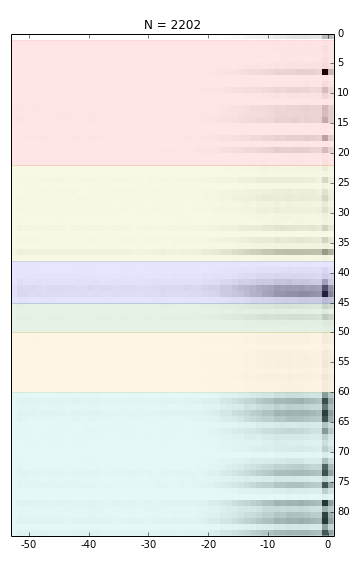 | 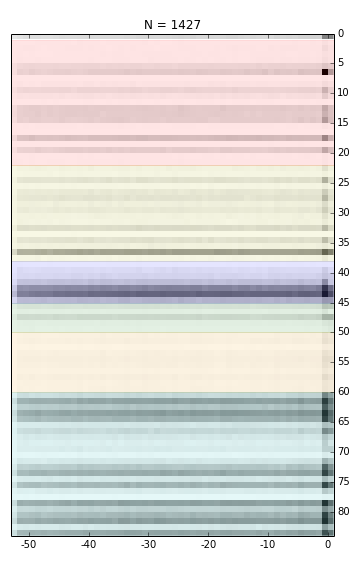 | 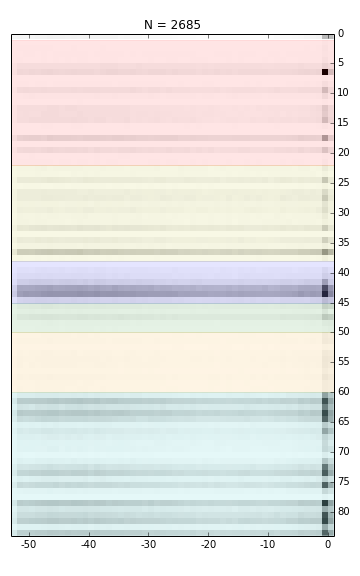 | 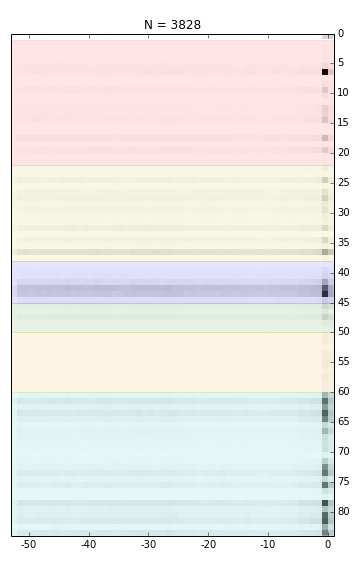 |
| Cluster 5 | Cluster 6 | Cluster 7 |  |
| 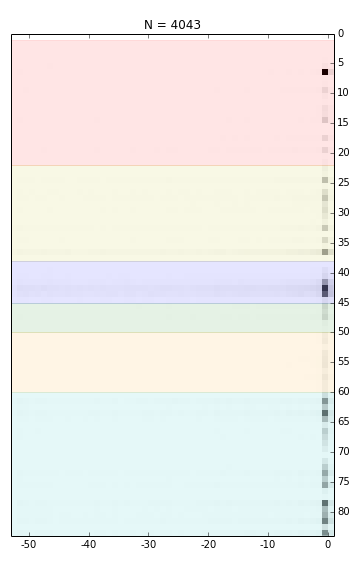 | 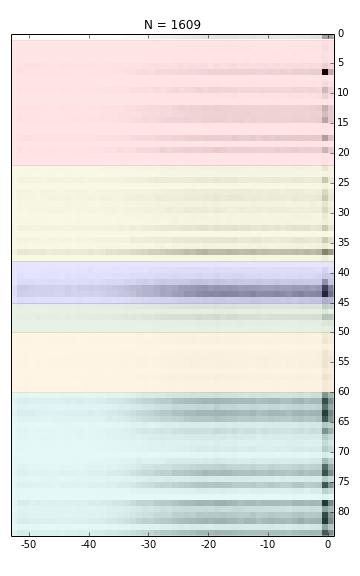 | 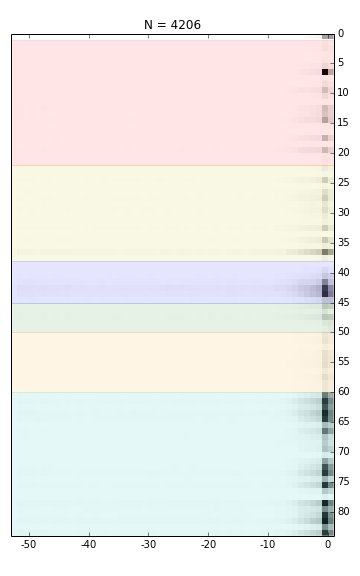 |  |

A7. Aggregated Temporal Images from the Clustering Result with 9 Clusters

| Cluster 1 | Cluster 2 | Cluster 3 |
| --- | --- | --- |
| 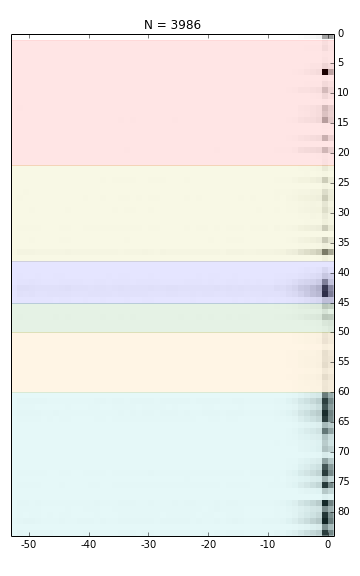 | 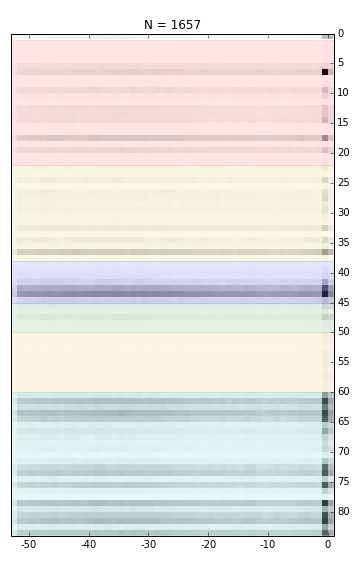 | 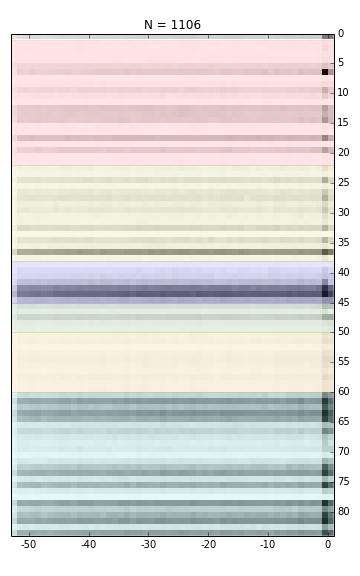 |
| Cluster 4 | Cluster 5 | Cluster 6 |
| 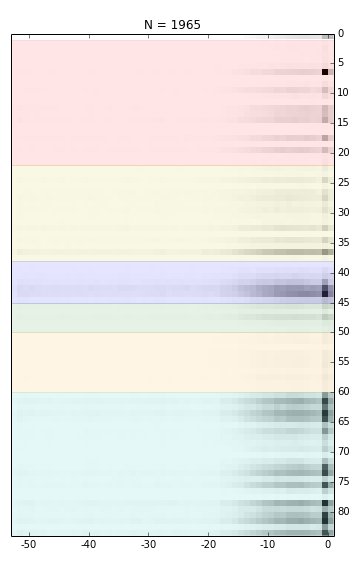 | 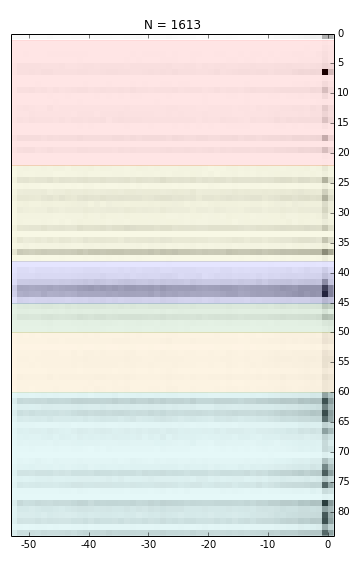 | 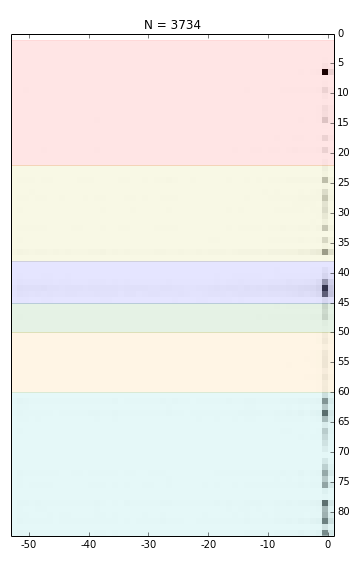 |
| Cluster 7 | Cluster 8 | Cluster 9 |
| 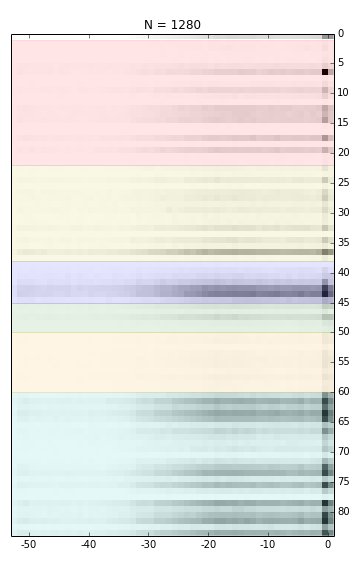 | 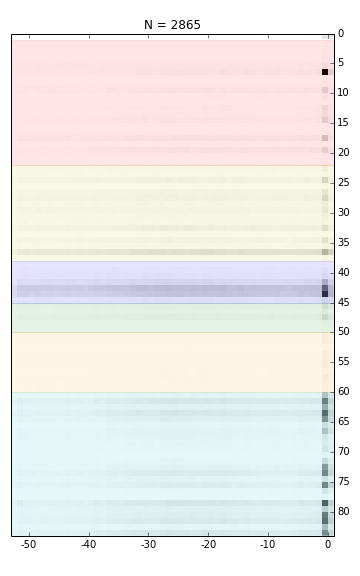 | 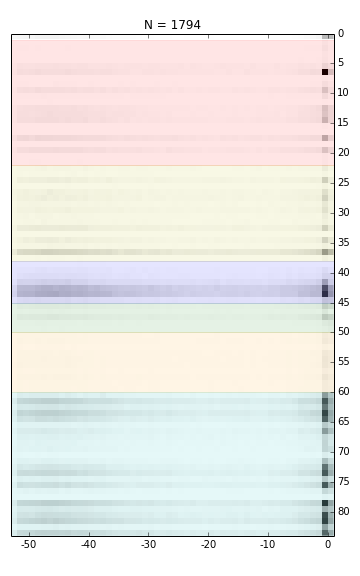 |

A8. Aggregated Temporal Images from the Clustering Result with 10 Clusters

| Cluster 1 | Cluster 2 | Cluster 3 | Cluster 4 |
| --- | --- | --- | --- |
| 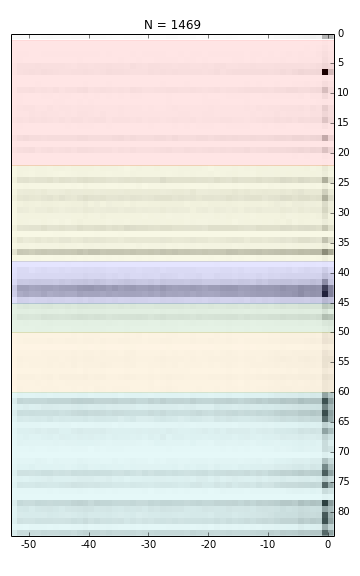 | 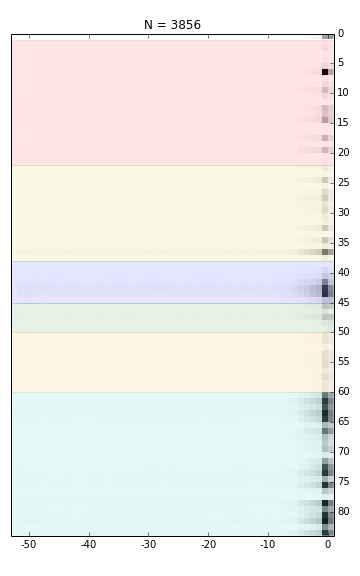 | 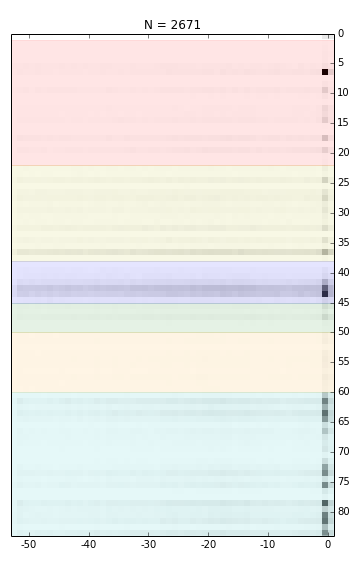 | 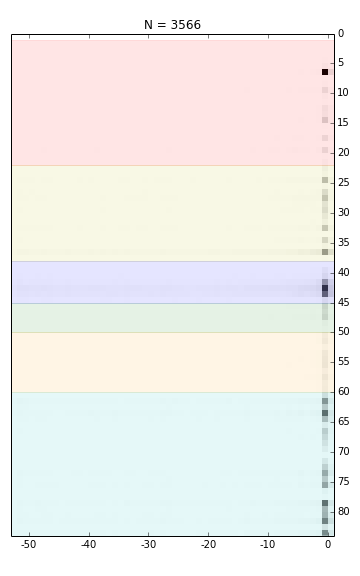 |
| Cluster 5 | Cluster 6 | Cluster 7 | Cluster 8 |
| 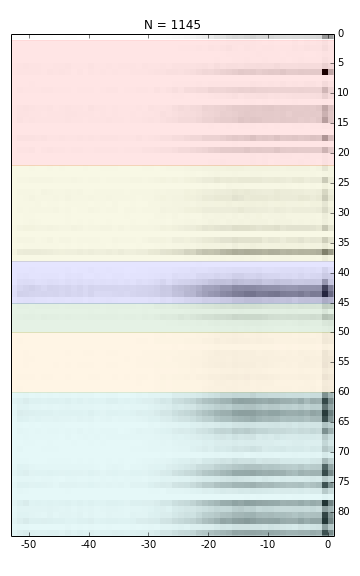 | 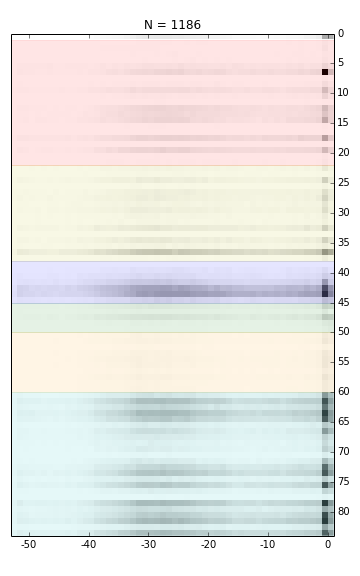 | 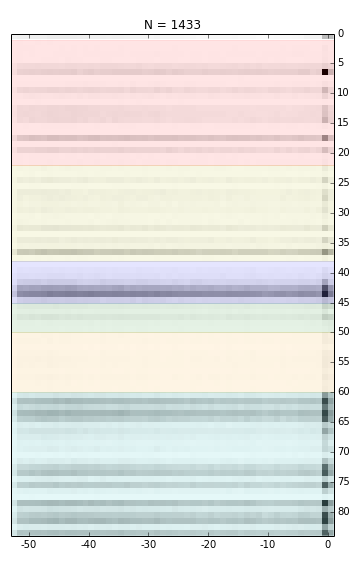 | 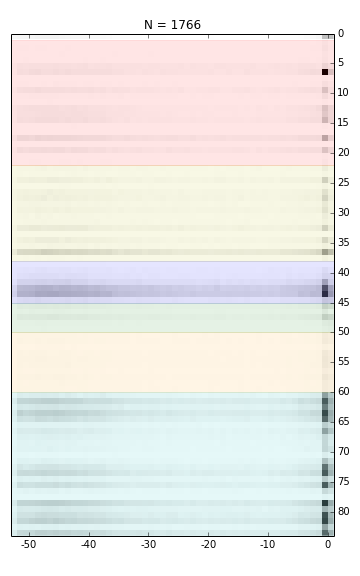 |
| Cluster 9 | Cluster 10 |  |  |
| 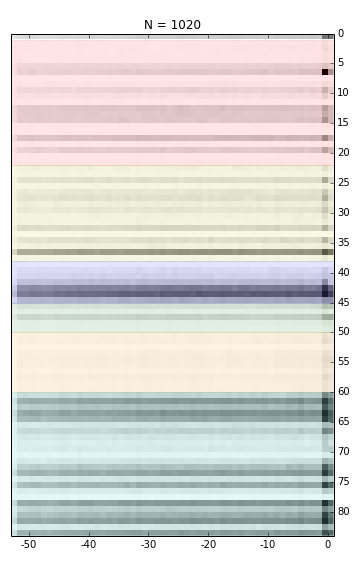 | 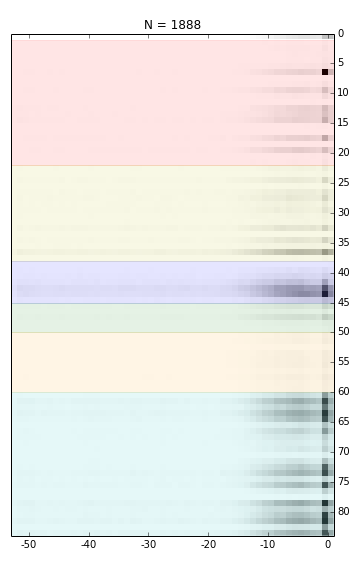 |  |  |

**Appendix IV Top Three Mental Disorders before Initial Bipolar Diagnosis**

| Rank | Cluster 1  N=4077 | Cluster 2  N=1386 | Cluster 3  N=1906 | Cluster 4  N=2105 | Cluster 5  N=3874 | Cluster 6  N=1617 | Cluster 7  N=3634 | Cluster 8  N=1401 |
| --- | --- | --- | --- | --- | --- | --- | --- | --- |
| 1 | Drug abuse, 42.5% | Depression, 70.5% | Depression, 57.6% | Depression, 57.3% | Drug abuse, 19.8% | Depression, 55.9% | Depression, 44.7% | Depression, 73.5% |
| 2 | Depression, 41.5% | Drug abuse, 65.9% | Drug abuse, 54.0% | Drug abuse, 54.8% | Depression, 17.9% | Anxiety, 47.1% | Drug abuse, 37.5% | Drug abuse, 72.9% |
| 3 | Anxiety, 29.4% | Anxiety, 55.5% | Anxiety, 50.1% | Anxiety, 44.0% | Anxiety, 15.3% | Drug abuse, 42.7% | Anxiety, 37.1% | Anxiety, 62.3% |

**Appendix V Top Ten Comorbid Conditions before Initial Bipolar Diagnosis**

| Rank | Cluster 1  N=4077 | Cluster 2  N=1386 | Cluster 3  N=1906 | Cluster 4  N=2105 | Cluster 5  N=3874 | Cluster 6  N=1617 | Cluster 7  N=3634 | Cluster 8  N=1401 |
| --- | --- | --- | --- | --- | --- | --- | --- | --- |
| 1 | Hypertension, 26.0% | Hypertension, 39.3% | Hypertension, 38.1% | Hypertension, 34.5% | Hypertension, 24.1% | Hypertension, 69.5% | Hypertension, 43.1% | Hypertension, 57.7% |
| 2 | Lipoid metabolism disorder, 18.7% | Disorder of back, 36.6% | Lipoid metabolism disorder, 37.8% | Disorder of back, 31.3% | Lipoid metabolism disorder, 22.7% | Lipoid metabolism disorder, 60.9% | Lipoid metabolism disorder, 41.8% | Disorders of joint, 53.3% |
| 3 | Disorder of back, 16.9% | Lipoid metabolism disorder, 36.1% | Disorder of back, 33.2% | Lipoid metabolism disorder, 28.0% | Disorder of back, 13.3% | Disorder of back, 47.4% | Disorder of back, 30.1% | Lipoid metabolism disorder, 51.7% |
| 4 | Disorders of joint, 13.9% | Disorders of joint,35.1% | Disorders of joint, 29.8% | Disorders of joint, 25.6% | Diseases of esophagus, 11.2% | Disorders of joint, 43.0% | Disorders of joint, 23.9% | Disorder of back, 50.0% |
| 5 | Diseases of esophagus, 10.9% | Diseases of esophagus, 24.0% | Diseases of esophagus, 21.6% | Diseases of esophagus, 16.5% | Diabetes, 10.9% | Diabetes, 38.8% | Diseases of esophagus, 19.3% | Disorders of refraction, 39.4% |
| 6 | Diabetes, 9.2% | Disorders of refraction, 21.9% | Disorders of refraction, 21.9% | Disorders of refraction, 14.7% | Disorders of joint, 9.6% | Disorders of refraction, 36.1% | Disorders of refraction, 18.7% | Diseases of esophagus, 36.9% |
| 7 | Overweight or obesity, 8.2% | Overweight or obesity, 19.6% | Overweight or obesity, 18.2% | Overweight or obesity, 13.3% | Overweight or obesity, 8.9% | Diseases of esophagus, 34.6% | Osteoarthrosis, 18.1% | Diseases Of oral cavity, 30.9% |
| 8 | Osteoarthrosis, 6.9% | Osteoarthrosis, 16.2% | Osteoarthrosis, 15.3% | Osteoarthrosis, 12.4% | Osteoarthrosis, 7.2% | Osteoarthrosis, 32.6% | Diabetes, 17.6% | Overweight or obesity, 30.9% |
| 9 | Viral hepatitis, 4.9% | Diseases Of oral cavity, 15.4% | Diseases Of oral cavity, 14.6% | Diabetes, 11.1% | Ischemic heart disease, 6.2% | Overweight or obesity, 31.3% | Overweight or obesity, 16.4% | Osteoarthrosis, 30.3% |
| 10 | Ischemic heart disease, 4.7% | Diabetes, 14.2% | Diabetes, 13.0% | Diseases Of oral cavity, 9.7% | Acquired hypothyroidism, 5.2% | Disorders of soft tissues, 24.2% | Disorders of soft tissues, 10.6% | Diabetes, 26.7% |

|  |  |
| --- | --- |
|  |  |
|  |  |
|  |  |

**Appendix VI Top Ten Symptoms by Cluster**

| Rank | Cluster 1  N=4077 | Cluster 2  N=1386 | Cluster 3  N=1906 | Cluster 4  N=2105 | Cluster 5  N=3874 | Cluster 6  N=1617 | Cluster 7  N=3634 | Cluster 8  N=1401 |
| --- | --- | --- | --- | --- | --- | --- | --- | --- |
| 1 | Depressed, 72.3% | Sleep, 99.7% | Sleep, 99.7% | Sleep, 97.2% | Depressed, 40.3% | Sleep, 98.2% | Sleep, 85.8% | Sleep, 100% |
| 2 | Sleep, 69.4% | Depressed, 98.1% | Suicidal, 95.7% | Depressed, 95.3% | Sleep, 31.5% | Tension, 94.4% | Depressed, 77.7% | Anxiety, 99.5% |
| 3 | Suicidal, 66.8% | Suicidal, 98.1% | Depressed, 95.6% | Suicidal, 93.0% | Hopeless, 30.9% | Depressed, 92.7% | Suicidal, 69.4% | Depressed, 99.4% |
| 4 | Hopeless, 57.8% | Anxiety, 97.5% | Anxiety, 94.1% | Anxiety, 88.7% | Nightmare, 25.6% | Anxiety, 90.1% | Anxiety, 69.1% | Suicidal, 99.2% |
| 5 | Anxiety, 54.5% | Anger, 92.3% | Anger 86.5% | Hopeless, 81.1 % | Suicidal, 24.5% | Suicidal, 87.2% | Tension, 66.5% | Anger, 97.9% |
| 6 | Nightmare, 46.7% | Hallucination, 92.0% | Hallucination, 84.6% | Hopeless, 81.1 % | Tension, 22.5% | Weakness, 82.0% | Hopeless 54.9% | Tension, 97.8% |
| 7 | Hallucination, 46.1% | Delusion, 91.6% | Delusion, 84.5% | Hallucination, 80.4% | Anxiety, 22.4% | Anger, 74.3% | Anger, 49.1% | Hallucination, 96.4% |
| 8 | Tension, 44.4% | Tension, 88.9% | Tension, 82.2% | Tension, 78.0% | Weakness, 13.5% | Tired, 72.5% | Hallucination, 48.1% | Delusion. 96.0% |
| 9 | Anger, 42.2% | Hopeless, 88.6% | Hopeless, 77.8 % | Anger, 78.0% | Tired, 12.5% | Hopeless, 72.5% | Weakness, 45.2% | Weakness, 93.6% |
| 10 | Delusion, 39.1% | Tired, 83.2% | Tired, 71.3% | Delusion, 74.6% | Hallucination, 12.0% | Hallucination, 71.1% | Nightmare, 44.4% | Hopeless, 92.8% |

|  |  |
| --- | --- |
|  |  |
|  |  |
|  |  |

Appendix VII Radar Chart for One-Year Outcomes Comparison among Clusters


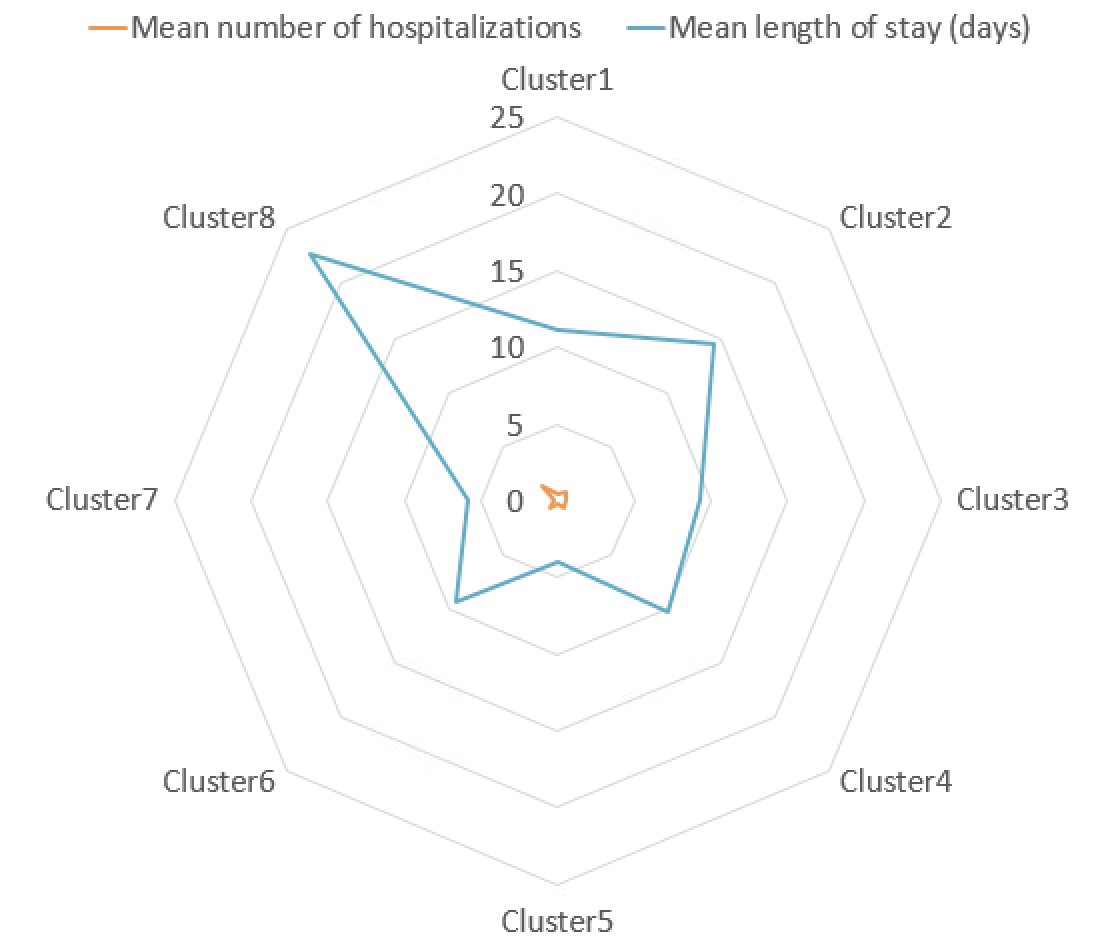

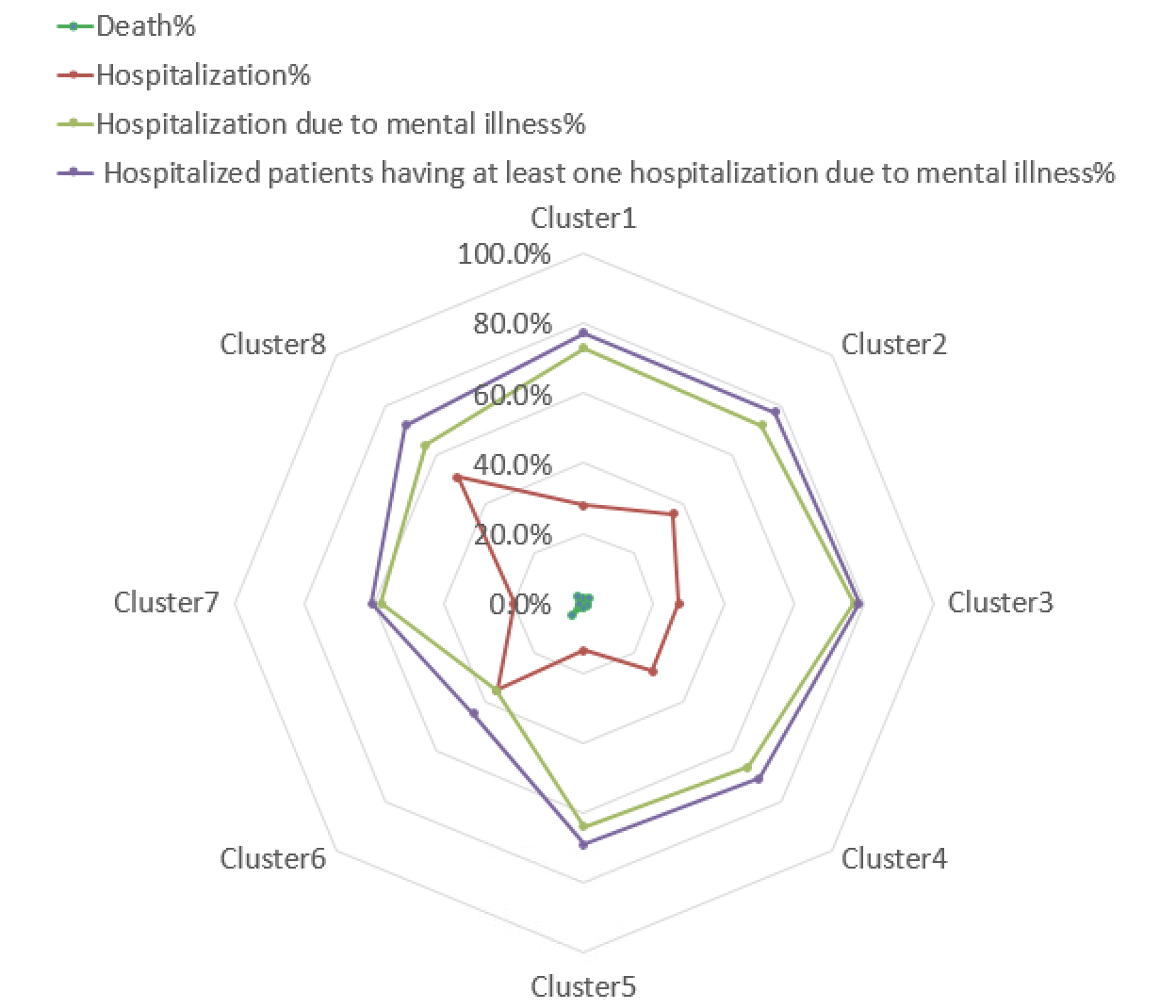

Supplement: Supplementary file 1 — Additional file 1. Appendix I-VII [file 40345_2023_298_MOESM1_ESM.docx]
